# Data on effluent toxicity and physicochemical parameters of municipal wastewater treatment plant using Daphnia Magna

**DOI:** 10.1016/j.dib.2018.06.076

**Published:** 2018-06-28

**Authors:** Fathollah Gholami-Borujeni, Fatemeh Nejatzadeh-Barandozi, Hamed Aghdasi

**Affiliations:** a Department of Environmental Health Engineering, Health Sciences Research Center, Addiction Institute, Mazandaran University of Medical Sciences, Sari, Iran; b Department of Horticulture, Faculty of Agriculture, Khoy Branch, Islamic Azad University, Khoy, Iran; c Department of Environmental Health Engineering, School of Health, Urmia University of Medical Sciences, Urmia, Iran

**Keywords:** Wastewater, Effluent, Acute toxicity, *Daphnia Magna*, Physicochemical parameter

## Abstract

Toxicity of Municipal Wastewater Treatment Plant Effluent (MWWTPE) was evaluated using bioassay with *Daphnia Magna* (*D. Magna*). Acute toxicity tests were performed on effluent samples of Urmia municipal wastewater treatment plant (Biolac system) according to the USEPA methods and 24, 48, 72, and 96 hr lethal concentration 50% (LC_50_) were calculated by application of Probit analysis. Also interrelationship between main effluent physicochemical parameters of wastewater (BOD_5_, COD, and TSS) and 24hr-LC_50_, were studied. Results showed that the effluent was safe to be discharged to the surface water in regard to physicochemical parameters and acute toxicity unit (TUa), according to the standards of Iranian Department of Environment (DOE). Relationship between effluent COD and 24hr-LC_50_ show that, increase in effluent COD resulted in increase in wastewater toxicity and there was not relationship between BOD_5_, TSS and toxicity of effluent.

**Specifications Table**

**Table:** 

Subject area	chemistry, biology
More specific subject area	Bio-assay of municipal wastewater treatment plants with D. Magna
Type of data	Table, image, figure
How data was acquired	Laboratory Bio-assay examination of Urmia Wastewater treatment plant effluent, BOD_5_, COD and TSS of MWWTP effluent. Electro-microscope equipped with camera was used to detection of *D.Magna* morphology changes
Data format	Raw and analyzed
Experimental factors	LC_50_ (Lethal Concentration 50%),(BOD_5_), (COD), (TSS)
Experimental features	For (QA/QC) of Bio- assay with *D. Magna*, All experiments were performed according to the standard method [1], [2]. Temperature of ambient test was measured during the experiments, culture medium of *D. Magna were prepared according to the guideline*[2], All experiments are repeated three times and average of data were report. It has been used as a standard test organism in U.S. Environmental Protection Agency (EPA), OECD, and International Organization for Standardization (ISO) standard protocols.
Data source location	MWWTPE of Urmia, Iran
Data accessibility	All data is included in this article

**Value of the data**

• These data provide the toxicity of Urmia Wastewater treatment plant effluent (Biolac lagoon) and relationships between BOD_5_, COD and TSS and LC_50_
• *Bio-Assay method with D. Magna may be applied to quality control of MWWTPE before discharge to the aquatic ambient as valuable tools for bio-monitoring of wastewater treatment plant effluent, as it is highly sensitive to pollutants*
• These data are valuable to researchers investigating LC_50_ related to the bio-chemical parameters of WWTPs effluents.

## 1 Data

**Fig. 1:**
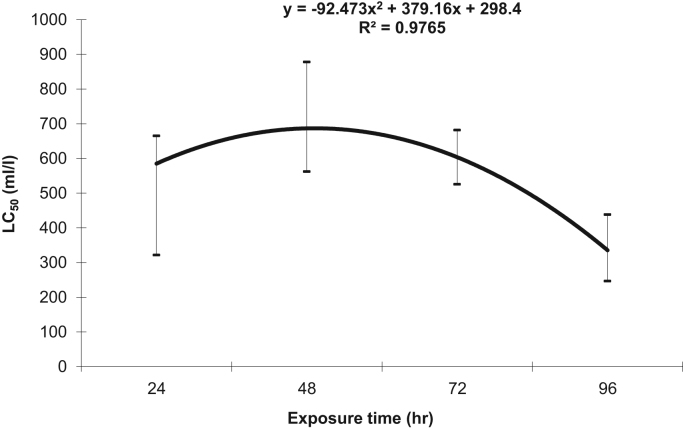
Acute toxicity Assay of Urmia Wastewater Treatment Plant effluent with *D. Magna*.

Chemical characteristics of Urmia wastewater treatment plant are presented in Table 1. The data of 24, 48, 72, and 96 h toxicity test of Urmia wastewater treatment plant effluent are presented at Fig. 1. In this Figure, lethal concentration 50 (LC_50_) of UWWTPE for 24, 48, 72, and 96 h exposure time are presented. This figure also presents the highest and lowest levels for 95% confidence. As well, a regression analysis was performed to examine possible correlations between exposure time and LC_50_. LC_50_ of Urmia wastewater treatment plant effluent was between 594 to 326 ml/l from exposure time of 24 to 96 h. Correlations between exposure time and LC_50_ show that there is non-linear regression between exposure time and LC_50_(R-squared is 0.97).

**Table 1:** Characteristics of Urmia wastewater treatment plant and comparison of effluent with standards published by Iran Department of Environment for discharge to surface waters.

	pH	BOD_5_ (mg/l)	COD (mg/l)	TSS(mg/l)	Nitrate (mg/l)	P (mg/l)
Untreated Wastewater	7.65±0.1	325±5	650±5	275±5	76±0.5	8±0.2
Treated Wastewater	8.01±0.2	20±2	40±1	15±0.5	13±0.2	2.5±0.1
Approved standards for discharge to surface waters Iranian DOE	6.5–8.5	30	60	40	50	6

**Fig. 2:**
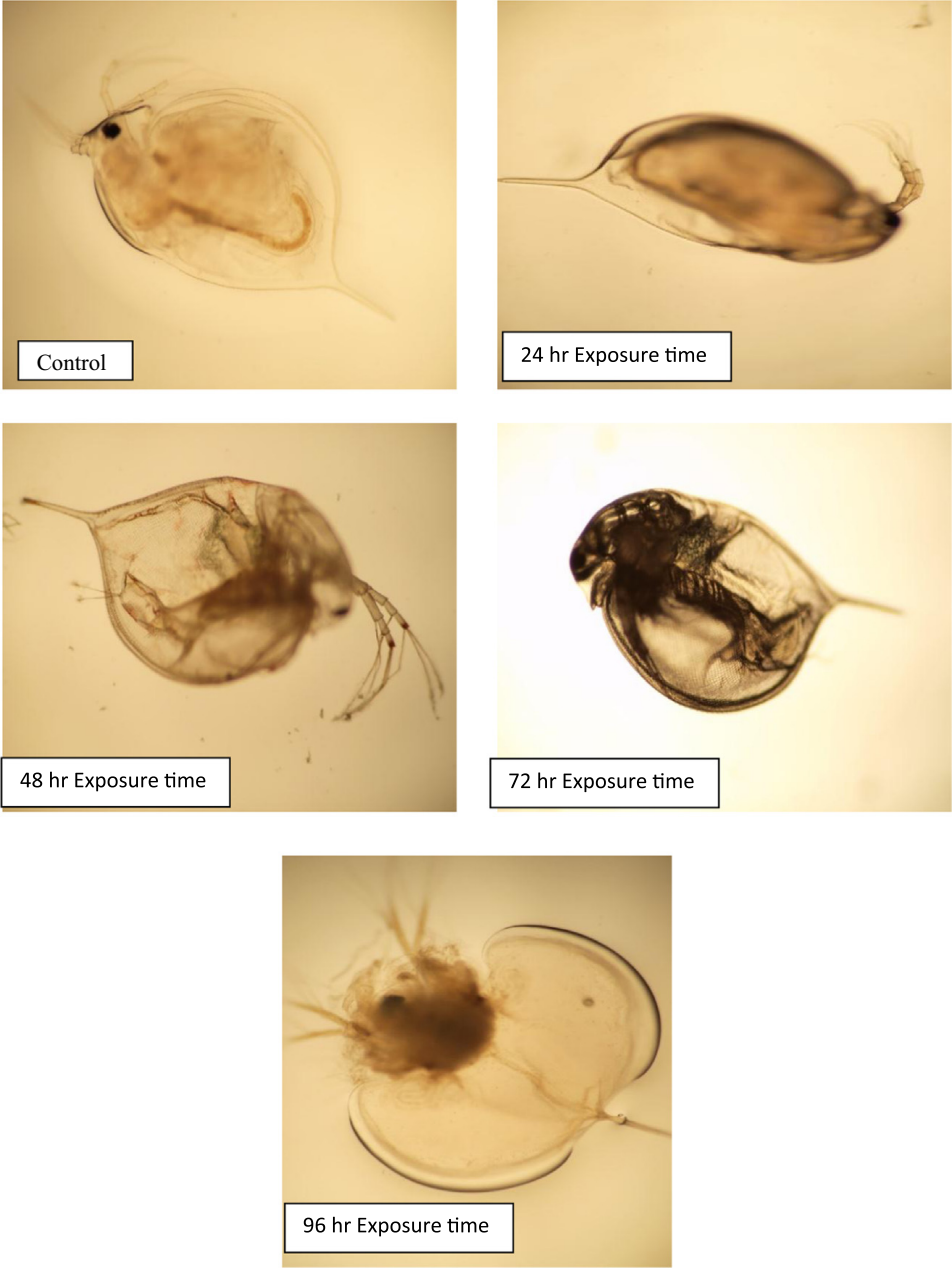
Light microscope images (40× magnifications) of *D. Magna* exposed to at different exposure time of Urmia wastewater treatment plant effluent.

**Fig. 3:**
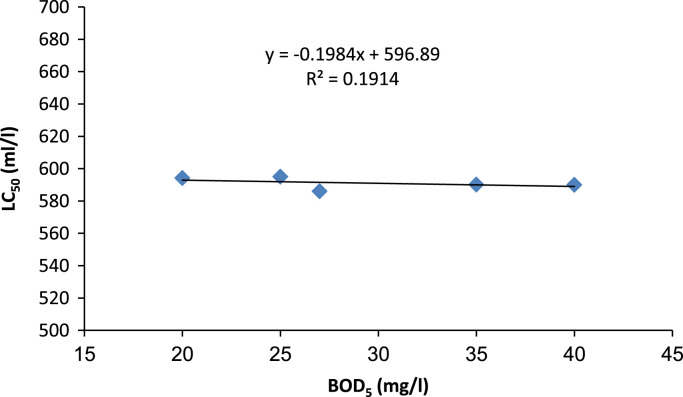
Interrelationship between concentration of BOD_5_ and toxicity of Urmia wastewater treatment plant effluent.

**Fig. 4:**
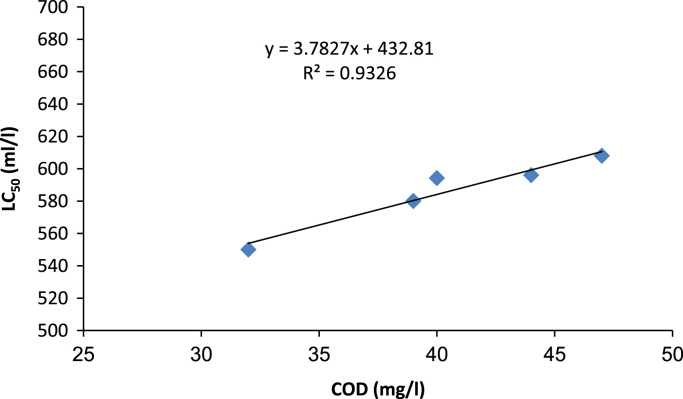
Interrelationship between concentration of COD and toxicity of Urmia wastewater treatment plant effluent.

**Fig. 5:**
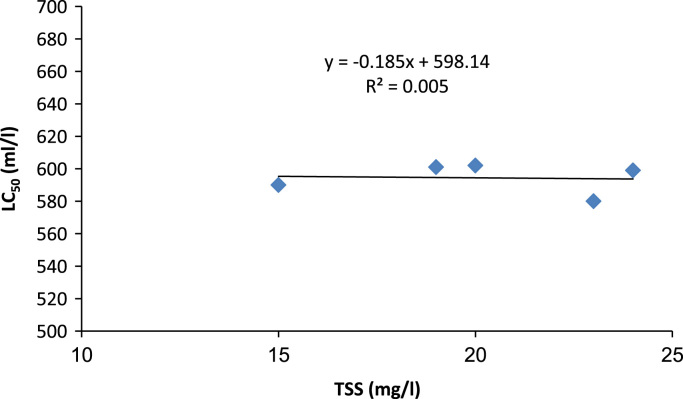
Interrelationship between concentration of TSS and toxicity of Urmia wastewater treatment plant effluent.

According to the light microscope images of *D. Magna,* large amounts of dark material were found in the gut tract of *D. Magna* after effluent wastewater exposure but not in the control. According to these images (Fig. 2), *D. Magna* after 96 h exposure time was destructed. The acute toxicity test were carried out to evaluate the inter-relationship between physicochemical parameters of effluent and toxicity of Urmia wastewater treatment plant effluent at various operational condition of wastewater treatment plant with different Biochemical Oxygen Demand (BOD_5_); (20–40 mg/l); Chemical Oxygen Demand; (COD) (30–50 mg/l); Total Suspended Solids (TSS); (15–25 mg/l) concentrations. These results are presented in Fig. 3, Fig. 4, Fig. 5. In addition, Relationship between BOD_5_ and 24h-LC_50_ are presented in Fig. 3. According to the results, there is no direct relationship between BOD_5_ concentration and acute toxicity of Urmia wastewater effluent (R-squared is 0.19). Relationship between COD and 24h-LC_50_ are presented in the Fig. 4. According to the results, there are linear and direct relationships between COD concentration and acute toxicity of Urmia wastewater effluent (R-squared is 0.93). Relationship between TSS and 24h-LC_50_ are presented in the Fig. 5. According to the results, there are not any linear and direct relationships between TSS concentration and acute toxicity of Urmia wastewater effluent (R-squared is 0.005) and this means that non settleable organic materials remain in effluent of wastewater have not acute toxicity effect on *D.Magna*. Total suspended solids (TSS) include all particles suspended in water which will not pass through a filter. After biological treatment of wastewater, some of the biological flocs (these flocs are mainly of biological origin) are not able to be settled in secondary sedimentation tanks and have not toxicity effects (Table 2).

**Table 2:** Acute toxicity classification of Urmia effluent wastewater treatment plant.

Exposure time	24 h	48 h	72 h	96 h
TUa	0.168	0.151	0.158	0.306
Classification [3]	No acute toxicity	No acute toxicity	No acute toxicity	No acute toxicity

## 2 Experimental design, materials, and methods

### 2.1 Wastewater treatment plant

Wastewater treatment system in the city of Urmia (One of the great western cities of Iran in west Azarbaijan province) that designed to serve 950,000 persons was selected to toxicity evaluation. Wastewater flow is about 77,000 m^3^/day mainly of domestic origin. This system consists of 2 mechanical coarse screening, Flow meter, Grit chamber, Biolac lagoon that have 5 part (Phosphor removal, Aeration, Sedimentation, Secondary aeration, Clarifier) and disinfection. In recent years to access the reclamation strategy for the Urmia Lake, these effluents were discharge to the lake. According to the Iranian Department of Environment (DOE) report this wastewater treatment plant effluents have standards for discharge to surface water.

### 2.2 Sampling

Samples were collected at different operational conditions from effluent of wastewater treatment plant in Jun 2015 based on the grab sampling, also known as a catch sampling that consists of a single sample taken at a specific time. All samples were collected and transport to laboratory according to the standards.

### 2.3 Propagation and culture of *D. Magna*

*D. Magna* was collected from a natural pond. Initially one of the isolated *D. Magna* was cultured. In the next step, the re-cultured *D.Magna* was used to prepare the final culture. For this reason, 100 ml of the final culture was poured into special bottles. Then, one single *D. magna* was added to each bottle. To support the growth of *D. Magna* during the day after the initial culture, one mg of dry yeast was added to each bottle every other day. Identification of *D.Magna* was carried out according to the US-EPA [4], [5].

In order to determine of LC_50_, different concentrations (sample and distilled water) of effluent were prepared using distilled water. The effluent samples were used at 20%, 30%, 40%, 50%, 60% and 70% and control samples according to the EPA recommendations for a toxicity test [4], [6]. Ten *D. Magna* were added to each dilution and the results of *D. Magna* mortality were recorded after exposure time. For each exposure time (24, 48, 72, and 96 h) this progress was repeated. Observations were made after 24, 48, 72, 96 h intervals.The results of experiments were acceptable only in cases where *D. Magna* in the blank tubes were observed to have a mortality rate of less than 10% [7]. It should be noted that temperature was checked regularly using a thermometer in the culture medium, Temperature of ambient test was measured during the experiments, culture medium of *D. Magna were prepared according to the guideline* [2], All experiments are repeated three times and average of data were report. An aerator pump was used to provide oxygen.

### 2.4 Statistical analysis

LC_50_ and their corresponding 95% confidence intervals were calculated by probit analysis (SPSS 16 version).Acute toxic unit (TUa) of effluent wastewater treatment plant was calculated as following equation [3], [8]:

(1) $$\mathrm{TUa}=100/{\mathrm{LC}}_{50}\%(v/v)$$

Toxicity classification is reported as follows:

No acute toxicity TUa< 0.4
Slight acute toxicity 0.4<TUa< 1
Acute toxicity 1≤TUa< 10
High acute toxicity 10≤TUa< 100
Very high acute toxicity100≥TUa

Each species endpoint per effluents solution sample was compared to the corresponding reference sample mean using a Students’*t* test. The difference was significant than p<0.05.

### 2.5 Physicochemical analysis

Effluent samples from the Urmia wastewater treatment plant were collected simultaneously in clean plastic cans and taken for physicochemical analysis following standard procedure [4]. Samples were preserved using preservatives as required, separately (for BOD_5_, COD, and TSS estimation).
