# Supplementary material for: Data on effluent toxicity and physicochemical parameters of municipal wastewater treatment plant using Daphnia Magna
Source: Data Brief. 2018 Jun 28;19:1837–43. doi: 10.1016/j.dib.2018.06.076 (PMC6141377; doi:10.1016/j.dib.2018.06.076)
Supplement: Supplementary file 1 — Supplementary material [file mmc1.doc]

Conflict of Interest and Authorship Conformation Form

Please check the following as appropriate:

- All authors have participated in (a) conception and design, or analysis and interpretation of the data; (b) drafting the article or revising it critically for important intellectual content; and (c) approval of the final version.
- This manuscript has not been submitted to, nor is under review at, another journal or other publishing venue.
- The authors have no affiliation with any organization with a direct or indirect financial interest in the subject matter discussed in the manuscript
- The following authors have affiliations with organizations with direct or indirect financial interest in the subject matter discussed in the manuscript:

Author’s name Affiliation

Fathollah Gholami-Borujeni Department of Environmental Health Engineering, Health Sciences Research Center, Mazandaran University of Medical Sciences, Sari, Iran

Fatemeh Nejatzadeh-Barandozi Department of Horticulture, Faculty of Agriculture, Khoy Branch, Islamic Azad University, Khoy, IRAN fnejatzadeh@yahoo.com

Hamed Aghdasi Department of Environmental Health Engineering, School of Health, Urmia University of Medical Sciences, Urmia, Iran env_eng1@yahoo.com
